# First case report of pustules associated with *Escherichia fergusonii* in the chinese pangolin (*Manis pentadactyla aurita*)

**DOI:** 10.1186/s12917-023-03622-3

**Published:** 2023-05-05

**Authors:** Fuyu An, Kai Wang, Shichao Wei, Hongmei Yan, Xuelin Xu, Jinqian Xu, Song Sun, Jiejian Zou, Fanghui Hou, Yan Hua

**Affiliations:** 1 Guangdong Provincial Key Laboratory of Silviculture, Protection and Utilization, Guangdong Academy of Forestry, Guangzhou, 510520 China; 2 College of Wildlife and Natural Protected Area, Northeast Forestry University, Harbin, 150040 China; 3 Guangdong Wildlife Rescue Monitoring Center, Guang Zhou, 510520 China; 4 Pangolin Conservation Research Center of National Forestry and Grassland Administration, Guang Zhou, 510520 China

**Keywords:** Chinese pangolin, Pustules, *Escherichia fergusonii*, Skin

## Abstract

**Background:**

*Escherichia fergusonii* is a common conditionally pathogenic bacterium that infects humans and animals. *E. fergusonii* has been reported to cause diarrhea, respiratory disease, and septicemia, but it is rarely reported to cause skin infections in animals. *E. fergusonii* has been isolated from the skin and muscular tissue of Chinese pangolin (*Manis pentadactyla aurita*). To date, there have been no reports of Chinese pangolins with clinical signs of skin diseases.

**Case presentation:**

This case report describes the clinical case of a subadult (bodyweight: 1.1 kg) female Chinese pangolin from wild rescue with pustules and subcutaneous suppurative infection due to *E. fergusonii* in the abdominal skin. Bacterial culture, Biochemical analysis, PCR and histopathology were utilized to identify the bacteria in the pustule puncture fluid and infected tissue. To the best of our knowledge, this is the first report of *E. fergusonii*-related pustules on a Chinese pangolin.

**Conclusion:**

This case report presents the first observed skin infection in a Chinese pangolin. *E. fergusonii* infection should be considered as a possible differential diagnosis of pustules and subcutaneous suppurative skin conditions in Chinese pangolins, and we also provide several recommendations for the diagnosis and treatment of this disease.

**Supplementary Information:**

The online version contains supplementary material available at 10.1186/s12917-023-03622-3.

## Background

*Escherichia fergusonii* belongs to the family *Enterobacteriaceae* [1, 2], which are common conditionally pathogenic bacteria that infect humans and animals. *E. fergusonii* has recently been shown to be responsible for wound infections, urinary tract infections, bacteremia, diarrhea, and pleural infections. *E. fergusonii* has been reported to cause diarrhea in animals such as in horses [3], reindeer [4], and ostriches [5], as well as causing respiratory disease and septicemia in pigs and sheep [6, 7]. However, few cases have been reported of *E. fergusonii* causing skin abscesses in animals or humans.

Here, we describe the first case of *E. fergusonii* infection in Chinese pangolin (*Manis pentadactyla aurita*).

## Case Report

**Fig. 1:**
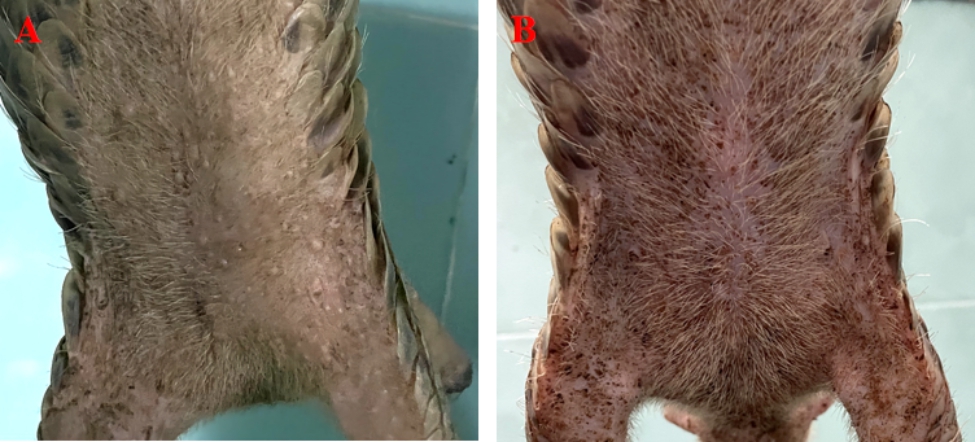
Clinical presentation of a bacterial infection of a Chinese pangolin: (A) The abdomen is covered with pustules of the various sizes, day 1; (B) Pustules disappeared from abdominal skin, day 7.

A subadult female Chinese pangolin (bodyweight: 1.1 kg) was found on the roadside and transported to Guangdong Wildlife Rescue Monitoring Center by local people. The pangolin was depressed, and the skin around her mouth and nares appeared pale and gray. The ventral skin from the mandible to the perineum was covered with pustules that ranged from 0.2 to 0.5 mm diameter (Fig. 1A). Moreover, there was skin ulceration on the right side of the abdominal genitalia, with subcutaneous suppurative infection, subcutaneous fat liquefaction, and necrosis.

Routine blood biochemical testing (Mindray, BC5000, automatic blood cell analyzer, ShenZhen, China) showed that the pangolin had a higher Phosphatase (PHOS) count and Mean Corpuscular Hemoglobin Concentration (MCHC) levels but lower amylase, Blood Urea Nitrogen (BUN), and Mean Corpuscular Volume (MCV) levels than those previously reported for Taiwanese pangolins (*M. P. pentadactyla*) (amylase: 62 U/L, reference interval [RI]: 148~538 U/L; BUN: 16 mg/dL, reference interval [RI]: 16.5~87 mg/dL; PHOS: 7.6 mg/dL, reference interval [RI]: 4.1~7.3 mg/dL; MCV: 56.4 f L, reference interval [RI]: 58.5~83.59 f L; MCHC: 366 g/L, reference interval [RI]: 31.3~38.6 g/L)[8, 9]. These test results suggest that this pangolin might be dehydrated.

We used gas anesthesia to calm the pangolin for subsequent physical examination and treatment. The pangolin was manually restrained for mask induction with isoflurane (100% isoflurane; ShangHai Yuyuan Instruments Co., Ltd., ShangHai, China) at a vaporizer setting of 4% in oxygen, delivered at 2 L/min through a Mapleson D nonrebreathing circuit until the animal’s muscle tone was relaxed (Superstar Medical Equipment, DM6A, Nan Jing, China) [10]. After induction, the animal was placed in sternal recumbency and maintained under anesthesia with 2% isoflurane delivered in oxygen at 2 L/min by a snug-fitting facemask.

**Fig. 2:**
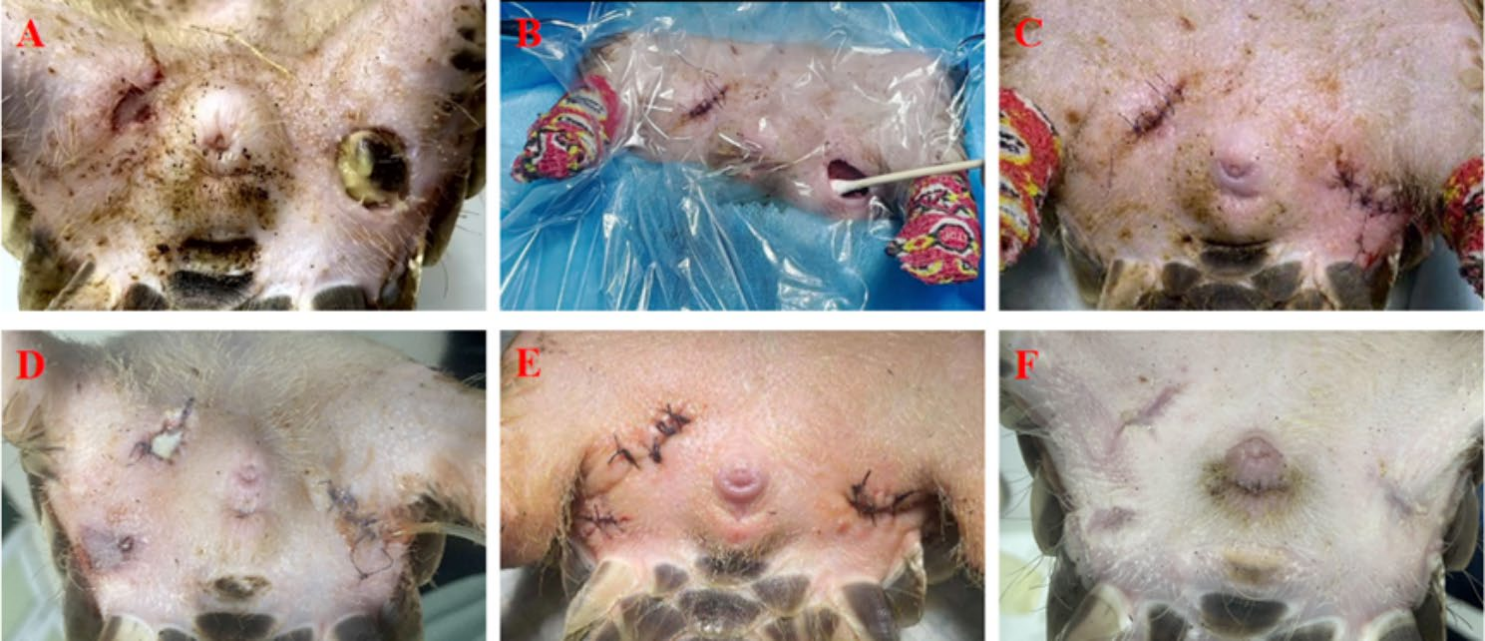
Clinical presentation of Chinese pangolin with subcutaneous abscesses in the course of treatmentcellulitis during treatment: 1st day (A); the removed necrotic connective tissue of 1st day (B); the wound was debrided, 1st day (C); wounds dehiscence with infection, 18th day (D); the wound was debrided secondly, 18th day (E); healed wound site, 58th day (F).

The thermistor probe was placed approximately 3 cm into the rectum to measure the pangolin’s rectal temperature (Mindray, uMEC12Vet, monitor, ShenZhen, China). The body temperature of the pangolin was 33.0 ~ 33.8℃during anaesthesia (the physiologic rectal temperature: 32.2 ~ 35.2℃) [11, 12]. On preliminary gross examination, in the center of the left wound, there was a 2 cm diameter scab (Fig. 2A). After removing the scab, there was a 2.5 cm diameter ulcer with inflammatory exudate on the skin of the left rear thigh and a 1 cm deep necrotic cavity below the scab, and filled with thick pasty yellow material (Fig. 2B). There were no additional macroscopic lesions of note. The full-thickness sample from the tissue removed was collected, fixed in 10% neutral buffered formalin, embedded in paraffin wax, sectioned at 4 μm, and stained with hematoxylin and eosin, and Giemsa stain for histopathological examination.

**Fig. 3:**
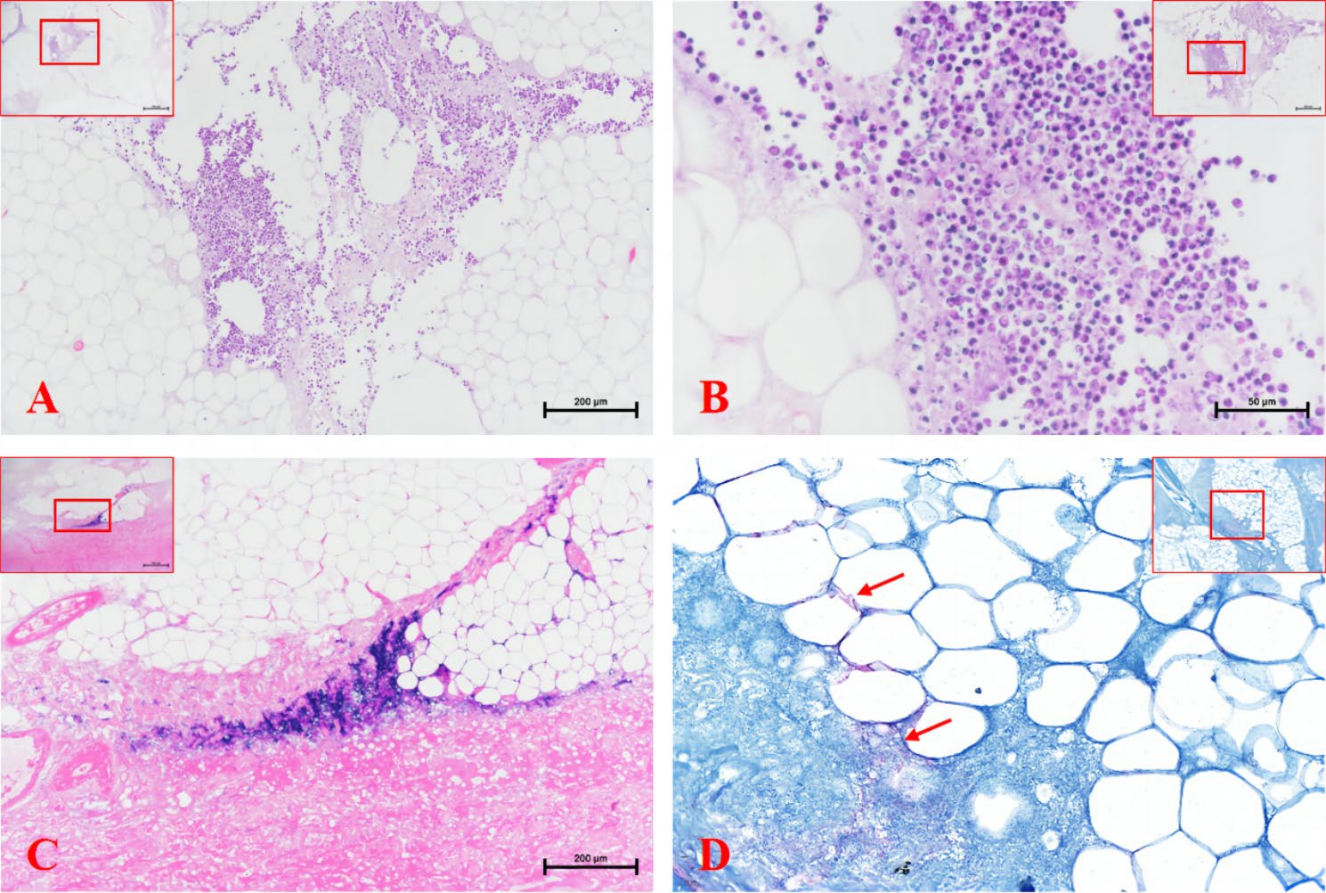
Histologic section of removed wound tissue (Figue 3-A to C is hematoxilin and eosin): (A) subcutaneous fat tissue with severe inflammatory infiltration by neutrophilic granulocytes; (B) higher magnification of (A); (C) subcutaneous necrotic tissue containing numerous bacterial colonies; (D) bacterial colonies in subcutaneous necrotic tissue seen under Giemsa stain

Histopathological examination of the tissues confirmed the grossly observed changes as necrotic cell debris, subcutaneous fat tissue with severe inflammatory infiltration by neutrophilic granulocytes, and subcutaneous necrotic tissue containing numerous bacterial colonies. Severe multifocal areas of necrosis admixed with minimal hemorrhage in the subcutaneous tissue and infiltration of numerous inflammatory cells in the adipose tissue and connective tissue were observed (Fig. 3A-B), numerous bacterial colonies were aggregated in the necrotic tissues of the dermis and subcutaneous tissue (Fig. 3C-D).

**Fig. 4:**
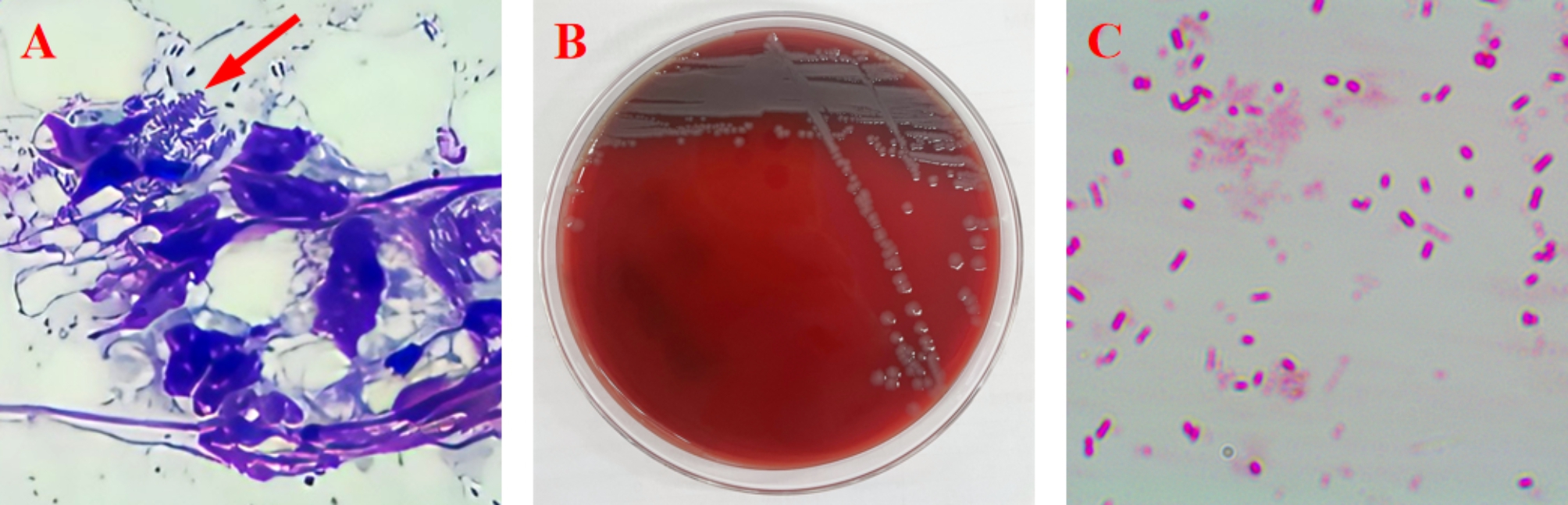
(A) Diff-quik staining revealing rod-shaped bacteria in the suppurative exsudate (10×100). (B) pale, translucent bacterial colonies cultured on blood agar. (C) Gram stain: The cultured bacteria was a rod-shaped, gram-negative, and generally motile (10×100)

Direct microscopic examination of the puncture fluid of the abscess and the pus of the wound showed no obvious parasites, such as vermiform mites. The Diff-quik staining of the pus by direct access showed a large number of rod-shaped bacterial cells (Fig. 4A). To identify the bacterial organisms, bacterial culture and PCR tests [13] were utilized to identify the bacteria in the lesions. Subcutaneous pus was collected from the ruptured wound, and subcutaneous pus was also collected from the abdominal pustules and plated on blood agar plates for bacterial culture (All puncture fluid samples were drawn from ruptured wound and subcutaneous pus using a 1 cc sterile syringe and 22-gauge needle). Pale, translucent bacterial colonies grew on blood agar (Fig. 4B).

The culture bacteria was a rod-shaped, gram-negative, and generally motile (Fig. 4C). The 16 S rRNA sequence amplified from the isolated strain was 99.72% identical to that of *E. fergusonii*. The product of the sequence consists of 1409 base-pairs. Nucleotide sequences of PCR products were analysed using Standard Nucleotide BLAST® NCBI Genomic Reference Sequences (https://blast.ncbi.nlm.nih.gov/Blast.cgi?PROGRAM=blastn&PAGE_TYPE=BlastSearch&LINK_LOC=blasthome). To further verify the cultured bacterial cells, we inoculated the bacterial cells into selective and differential media and biochemical analysis. Biochemical analysis of *E. fergusonii*-like isolates was carried out using the Bacterial biochemical analysis tube (QingDao Hopebio Co., Ltd., QingDao, China). All biochemical tests confirmed the biochemical characteristics of *E. fergusonii*. They are positive for lysine decarboxylase and ornithine decarboxylase. They ferment adonitol, cellobiose, L-rhamnose, D-arabinitol, D-mannitol and D-xylose and ferment glucose with gas production. They are negative for growth in Dulcitol Semisolid Agar, esculin hydrolysis, fermentation of lactose, D-sorbitol, and raffinose. The bacterium was identified as *E. fergusonii* by amplification of conserved genes with reference to the primers used for the identification of *E. fergusonii* by Lindsey et al. (2017) [13]. The details of the primers are shown in the supplementary materials (Tables S1–S2).

Forty antibiotics were chosen for testing (Oxoid, Basingstoke, UK): penicillin G, ampicillin, amoxicillin/clavulanate, ticarcillin/clavulanate, oxacillin, amoxicillin, gentamicin, amikacin sulfate, streptomycin, doxycycline, tetracycline, azithromycin, erythromycin, chloramphenicol, clindamycin, enrofloxacin, marbofloxacin, ciprofloxacin, norfloxacin, ofloxacin, nitrofurantoin, cefazolin, cefalexin, cefuroxime, cefoxitin, ceftiofur, cefotaxime, ceftriaxone, cefovecin, cefquinome, cefoperazone/sulbactam, sulfanilamide + trimethoprim, meropenem, imipenem, aztreonam, vancomycin, polymyxin B, rifampicin, lincomycin, and metronidazole. Results were interpreted in accordance with CLSI criteria. ATCC35469 was used as a control. The results of the drug resistance test showed that this strain is resistant to 38 antibiotics and only showed intermediately resistant to amikacin sulfate and imipenem sulfate. Details of the drugs used and the results of the drug sensitivity tests are given in the supplementary material (Tables S3).

On the first day of treatment, simple cleaning and disinfection of the abdominal skin were performed to avoid pustule rupture and secondary infections. Debridement and irrigation were performed on the wound, and a drainage tube was placed for drainage. F10 Germicidal Barrier Ointment (Health and Hygiene (Pty) Ltd., Gauteng, South Africa) was wiped on the surface of the wound before skin closure with a 3 − 0 monofilament nylon suture (Fig. 2C). The pangolin was injected with antibiotics (amikacin sulfate 4.4 mg/kg) administered subcutaneously twice a day (Jilin Huamu Animal Health products Co., Ltd., Changchun, China), and butorphanol was used for postoperative analgesia (0.2 mg/kg, once per day) (Butormin, Holliday-Sott S.A., BA, Argentina), and the wound was wiped with F10 Germicidal Barrier Ointment daily from 1st day to 7th day.

On the 7th day of treatment, most of the skin abscesses on the abdomen had disappeared from the pangolin (Fig. 1B). However, the suture line on the pangolin’s right side of the abdominal wound ruptured. After the pangolin was anesthetized again, the wounds were debrided and sutured. On the 18th day of treatment, we found that the suture line of the wound had ruptured again, and there was new subcutaneous hemorrhage and necrosis of the skin below the pangolin’s right side of the abdominal wound (Fig. 2D).

After the pangolin was anesthetized again, all wounds were debrided and sutured (Fig. 2E). On the 28th day of treatment, the suture line on both sides of the pangolin’s abdominal wound ruptured. The pangolin would scratch the wound, causing the stitches to open and the wound to tear, which we observed through the monitoring system in the nursing room. Drawing on our previous wildlife nursing experience, we decided to leave the wound open, and we transferred the animal to a wooden box (The box size: 60 × 50 × 50cm), which was small, dark, and breathable. The wooden box is spread with soft and absorbent towels. Amikacin sulfate (4.4 mg/kg) was administered subcutaneously twice a day from 28th day to 35th day. The wound was washed with chlorhexidine solution daily, wiped with sterile gauze, and then covered with Bletilla ointment; the bedding materials were replaced in a timely fashion to ensure environmental hygiene from 28th day to 58th day.

At the same time, the feed formula of the pangolin was adjusted to provide termites as the main material, with the addition of other appropriate insects such as silkworm (*Bombyx mori* ) pupa, mealworms and earthworms, as well as complex vitamin B (10U/kg, once per day) (Guangdong Hengjian Pharmaceutical Co., Ltd., Guangzhou, China), vitamin A (3000IU, once per day) (Qingdao Shuangjing Pharmaceutical Co., Ltd., Qingdao, China), vitamin E (100IU, once per day) (Qingdao Shuangjing Pharmaceutical Co., Ltd., Qingdao, China), vitamin C (22 mg/kg, once per day) (Guangdong Hengjian Pharmaceutical Co., Ltd., Guangzhou, China) and zinc (10 mg/kg, once per day) (Shandong Yikang Pharmaceutical Co., Ltd. Tengzhou, China) during the whole treatment period (The amount of various vitamins added is similar to that used by small animals and known pangolins) [14–16]. On the 58th day of treatment, the wound was fully healed (Fig. 2F).

## Discussion and conclusions

At present, our understanding of pangolins’ biological characteristics and diseases is still limited [17]. The methods of body condition examination and disease diagnosis refer to the clinical diagnosis and treatment techniques developed for other small mammals. The case reports of diseases involving pangolins have only been reported in traumatic infection [18, 19], vitamin A deficiency [20], parasitic infection [21] and canine parvovirus [22, 23] infection. Here, we report the diagnosis and treatment of severe pustules on the skin of a Chinese pangolin infected with *E. fergusonii*. Our methodologies and results will be a great reference for wildlife veterinarians and rehabilitators and will contribute to increasing the successful rescue of pangolins.

At present, it has been reported that the main pathogenic bacteria isolated from pangolins are *E. coli*, *Klebsiella pneumoniae*, *Proteus vulgaris*, *Streptococcus faecalis*, and *Staphylococcus sp.* [24, 25]. The results of bacterial culture, and 16 S rDNA sequence amplification, and PCR test provided confirmatory evidence that the lesions in the skin of this pangolin were associated with *E. fergusonii.*

In 1985, *E. fergusonii* was proposed as a new species of the genus *Escherichia* in the family *Enterobacteriaceae* [1, 2]. *E. fergusonii* has been reported to cause diarrhea in animals such as horses [3], reindeer [4], and ostriches [5], as well as respiratory disease and septicemia in pigs and sheep [6, 7]. Reports of skin diseases caused by *E. fergusonii* are rare in animals, but are described for human patients [1, 6, 26]. To our knowledge, this is the first reported case of skin pustules and abscesses in the Chinese pangolin caused by *E. fergusonii*.

Multidrug-resistant *E. fergusonii* strains and Extended-Spectrum β-Lactamases (ESBL) producing isolates have been reported repeatedly in human patients [27–29]. *E. fergusonii* were shown to be resistant to many drugs, including ciprofloxacin, ceftriaxone, amoxicillin/clavulanate, ampicillin, and polymyxin making susceptibility testing essential to guide therapy. Out of 40 different antibiotics the strain isolated from this pangolin was only sensitive to imipenem sulfate and amikacin sulfate. The latter was successfully used for treatment.

As the pangolin’s medical history is unknown, we considered the species’ predation habits, the onset season and the age and rescue site of the pangolin. These factors combined with the clinical manifestations of the pustular infection in the bare leaking skin of the abdomen suggest parasitic bites or ant bites, leading to secondary skin bacterial infection. Skin Mites infection has been reported to cause suppurative dermatitis on the skin of pangolins by Khatri-Chhetri et al., which also suggests that we should consider mites infection as one of top list differential diagnoses for such symptoms in pangolins [30, 31]. Although microscopic examination of the curetted skin revealed no parasites, it did not exclude the possibility of ant bites causing skin damage and subsequent infection by *E. fergusonii*, but it is difficult to investigate these pathogenic causes.

Based on the data from rescued Chinese pangolin trauma cases from zoos and wildlife rescue centers around the world [18, 19, 32], wound management not only requires intensive surgical debridement, topical treatment, and bandaging but also requires a reasonable nursing management for healing. Such as three traumatic cases of pangolin that had the modified Choukroun’s platelet-rich fibrin technique (MC-PRF) incorporated into the wound care plan in Taiwan [19]. This technique provides faster wound healing times than traditional wound management practices, and Platelet-rich plasma (PRP) might provide early protection against bacterial contamination, but detailed clinical trials are needed to evaluate the true efficacy of this protocol in pangolins, so we did not use of MC-PRF in this case. A series of Taiwan pangolins case reports from Taipei Zoo Rescue Center demonstrated that precise surgery and careful nursing shorten the time of wound healing, thereby reducing medical intervention times to increase the success rate [19].

In our study, it was observed that the activity frequency of injured pangolins is increased due to stress, such as wound pain and scratching itself, resulting in wound tearing after the first and second operations. Therefore, a quiet, hygienic, and narrow space was provided to reduce the pangolin’s mobility and prevent additional wound tearing, which could cause a secondary infection. Pangolin wound management not only needs to make a reasonable plan for the wound but also needs to make appropriate adjustments of the diet. Proper addition of vitamin C and other vitamins to the feed could improve immunity, reduce the stress response, and speed up wound healing [20, 33]. Vitamin A deficiency has been reported to cause significant eye symptoms, anorexia, and leading to reduced resistance to bacteria in captive pangolins [20]. Low levels of vitamin E have been reported in chronic wound patients [34, 35]. Appropriate vitamin A and E supplements can improve the above symptoms, and increases the inflammatory response, angiogenesis, and reparative collagen synthesis in incisional wounds [34, 36]. Vitamin B complex and Zinc helps to promote cell proliferation, maintain healthy skin and muscle tone, support and increase metabolic rate [37, 38]. In addition, it has been reported that giving certain doses of meloxicam to pangolins can relieve their anxiety caused by pain and alleviate inflammation [14]. Considering that this pangolin might be dehydrated, butorphanol was used for postoperative pain management to reduce the risk of nephrotoxicity [39, 40]. This case report describes the first successful treatment of a Chinese pangolin with a suppurative skin infection caused by *E. fergusonii*. Meanwhile, *E. fergusonii* infestation should be considered as a possible differential diagnosis of pustules and subcutaneous suppurative skin conditions in Chinese pangolins. As an opportunistic pathogen, *E. fergusonii* has highly resistant to the antibiotics used in this case, and future research is needed to monitor the antimicrobial resistance in wild rescue pangolins, which will help to establish strategies for prevention.

## Electronic supplementary material

Below is the link to the electronic supplementary material.

Supplementary Material 1

## Data Availability

The 16 S nucleotide sequences of *E. fergusonii* generated in the present study have been deposited in GenBank database under the accession numbers NR_027549.1.

## Abbreviations

*E. fergusonii*: *Escherichia fergusonii*
*M. p. pentadactyla*: *Manis pentadactyla pentadactyla*
