# Supplementary material for: First case report of pustules associated with Escherichia fergusonii in the chinese pangolin (Manis pentadactyla aurita)
Source: BMC Vet Res. 2023 May 5;19:69. doi: 10.1186/s12917-023-03622-3 (PMC10163759; doi:10.1186/s12917-023-03622-3)
Supplement: Supplementary file 1 — Supplementary Material 1 [file 12917_2023_3622_MOESM1_ESM.docx]

Supplementary Material

**The** **16S rRNA sequences of isolated bacteria**

**5’-3’:**

CACATGCAGTCGAACGGTAACAGGNATCAGCTTGCTGATTNGCTGACGAGTGGCGGACGGGTGAGTAATGTCTGGGAAACTGCCTGATGGAGGGGGATAACTACTGGAAACGGTAGCTAATACCGCATAANGTCGCAAGACCAAAGAGGGGGACCTTCGGGCCTCTTGCCATCGGATGTGCCCAGATGGGATTAGCTAGTAGGTGGGGTAACGGCTCACCTAGGCGACGATCCCTAGCTGGTCTGAGAGGATGACCAGCCACACTGGAACTGAGACACGGTCCAGACTCCTACGGGAGGCAGCAGTGGGGAATATTGCACAATGGGCGCAAGCCTGATGCAGCCATGCCGCGTGTATGAAGAAGGCCTTCGGGTTGTAAAGTACTTTCAGCGGGGAGGAAGGGAGTAAAGTTAATACCTTTGCTCATTGACGTTACCCGCAGAAGAAGCACCGGCTAACTCCGTGCCAGCAGCCGCGGTAATACGGAGGGTGCAAGCGTTAATCGGAATTACTGGGCGTAAAGCGCACGCAGGCGGTTTGTTAAGTCAGATGTGAAATCCCCGGGCTCAACCTGGGAACTGCATCTGATACTGGCAAGCTTGAGTCTCGTAGAGGGGGGTAGAATTCCAGGTGTAGCGGTGAAATGCGTAGAGATCTGGAGGAATACCGGTGGCGAAGGCGGCCCCCTGGACGAAGACTGACGCTCAGGTGCGAAAGCGTGGGGAGCAAACAGGATTAGATACCCTGGTAGTCCACGCCGTAAACGATGTCGACTTGGAGGTTGTGCCCTTGAGGCGTGGCTTCCGGAGCTAACGCGTTAAGTCGACCGCCTGGGGAGTACGGCCGCAAGGTTAAAACTCAAATGAATTGACGGGGGCCCGCACAAGCGGTGGAGCATGTGGTTTAATTCGATGCAACGCGAAGAACCTTACCTGGTCTTGACATCCACGGAAGTTTTCAGAGATGAGAATGTGCCTTCGGGAACCGTGAGACAGGTGCTGCATGGCTGTCGTCAGCTCGTGTTGTGAAATGTTGGGTTAAGTCCCGCAACGAGCGCAACCCTTATCCTTTGTTGCCAGCGGTCCGGCCGGGAACTCAAAGGAGACTGCCAGTGATAAACTGGAGGAAGGTGGGGATGACGTCAAGTCATCATGGCCCTTACGACCAGGGCTACACACGTGCTACAATGGCGCATACAAAGAGAAGCGACCTCGCGAGAGCAAGCGGACCTCATAAAGTGCGTCGTAGTCCGGATTGGAGTCTGCAACTCGACTCCATGAAGTCGGAATCGCTAGTAATCGTGGATCAGAATGCCACGGTGAATACGTTCCCGGGCCTTGTACACACCGCCCGTCACACCATGGGAGTGGGTTGCAAAAGAAGTAGGTAGCTTAACCTTCGGGAGGGCG **(1409bp)**

# Supplementary Tables

**Table S1.** **The PCR primers, reaction system and cycling conditions of 16s rRNA.**

| **Item** | **Primer (5’-3’)** | **Reaction system** | **Cycling conditions** | **Lenrth (bp)** |
| --- | --- | --- | --- | --- |
| 16s rRNA | 27F:AGAGTTTGATCCTGGCTCAG  1492R:TACGGCTACCTTGTTACGACTT | 1.5 μL of each primer, 2ｘTapPCR Master Mix 20μL, ddH2O 27μL | one cycle at 95 °C for 5 min, 95 °C for 30 s, 56 °C for 30 s, and 35 cycles of 72 °C for 90 s, and one final cycle at 72 °C for 10 min. | 1400-1500 |

**Table S2. The PCR primers, reaction system and cycling conditions of** **PCR test for the *Escherichia fergusonii*.**

| **Primer (5’ to 3’)** | **Reaction system** | **Cycling conditions** | **Lenrth (bp)** |
| --- | --- | --- | --- |
| EF_F：5′-AGATTCACGTAAGCTGTTACCTT-3′  EF_R：5′-CGTCTGATGAAAGATTTGGGAAG-3′ | 1.5 μL of each primer, 2ｘTapPCR Master Mix 20μL, ddH2O 27μL | one cycle at 95 °C for 10 min, 92 °C for 1 min, 57 °C for 1 min, and 30 cycles of 72 °C for 30 s, and one final cycle at 72 °C for 5 min. | 575 |

**Table S3. Results of antibiotic and drug sensitivity tests used for drug susceptibility testing.**

| **Number** | **Antibiotics** | **Dosage** | **Susceptibility test** |
| --- | --- | --- | --- |
| 1 | Penicillin G | 10units | R |
| 2 | Ampicillin | 10μg | R |
| 3 | Amoxicillin/clavulanate | 30μg | R |
| 4 | Ticarcillin/clavulanate | 75μg | R |
| 5 | Oxacillin | 1μg | R |
| 6 | Amoxicillin | 10μg | R |
| 7 | Gentamicin | 10μg | R |
| 8 | Amikacin sulfate | 30μg | I |
| 9 | Streptomycin | 10μg | R |
| 10 | Doxycycline | 30μg | R |
| 11 | Tetracycline | 30μg | R |
| 12 | Azithromycin | 15μg | R |
| 13 | Erythromycin | 15μg | R |
| 14 | Chloramphenicol | 30μg | R |
| 15 | Clindamycin | 2μg | R |
| 16 | Enrofloxacin | 5μg | R |
| 17 | Marbofloxacin | 5μg | R |
| 18 | Ciprofloxacin | 10μg | R |
| 19 | Norfloxacin | 10μg | R |
| 20 | Ofloxacin | 5μg | R |
| 21 | Nitrofurantoin | 300μg | R |
| 22 | Cefazolin | 30μg | R |
| 23 | Cefalexin | 30μg | R |
| 24 | Cefuroxime | 5μg | R |
| 25 | Cefoxitin | 30μg | R |
| 26 | Ceftiofur | 30μg | R |
| 27 | Cefotaxime | 30μg | R |
| 28 | Ceftriaxone | 30μg | R |
| 29 | Cefovecin | 30μg | R |
| 30 | Cefquinome | 5μg | R |
| 31 | Cefoperazone/Sulbactam | 30μg | R |
| 32 | Sulfanilamide+Trimethoprim | 25μg | R |
| 33 | Meropenem | 10μg | R |
| 34 | Imipenem | 10μg | I |
| 35 | Aztreonam | 30μg | R |
| 36 | Vancomycin | 30μg | R |
| 37 | Polymyxin B | 300IU | R |
| 38 | Rifampicin | 5μg | R |
| 39 | Lincomycin | 10μg | R |
| 40 | Metronidazole | 5μg | R |

Forty antibiotics were chosen for testing (Oxoid, Basingstoke, UK).

Legend: S= sensitive, I = intermediate, R= resistant. Testing was performed according to CLSI.

# Supplementary Figure


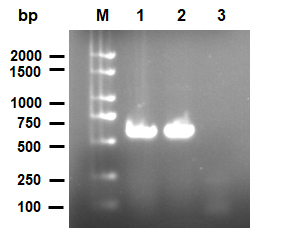


**Figure 1.** Agarose gel electrophoresis of specific primers from the *Escherichia fergusonii* PCR.

M: Low DNA Mass Ladder. 1: Standard strains. 2: *Escherichia fergusonii*-like bacteria (575 bp). 3: No DNA, water control.

**PCR amplification and electrophoresis**

Isolated DNA samples were amplified using Veriti® Thermal Cycler (Applied Biosystems, Thermo Fisher, Waltham, USA) and PCR kits supplied by HotStarTaq Master Mix Kit (Vazyme Biotech Co., Ltd., Nanjing, China). The PCR primers, reaction system and cycling conditions of PCR test for the *Escherichia fergusonii* (Table S2). PCR products (4 μl each) or 2 μl of Low DNA Mass Ladder (Vazyme Biotech Co., Ltd., Nanjing, China) were diluted in 2 μl of 5× bromophenol blue dye and electrophoresed on a 2% agarose gel prepared with TAE (40 mM Tris-acetate, pH 8.3, 1 mM EDTA) buffer. Electrophoresis was performed at a constant voltage of 100 V for 45 min and the agarose gel was stained with GelRed™ Nucleic Acid Gel Stain (VWR, Radnor, USA). Agarose gels were imaged under UV light using the Gel Doc™ XR+ system (Tanon science & technology Co., Ltd., Shanghai, China) and analyzed for band sizes.
